# Bridging the Gap Between Prosody and Pragmatics: The Acquisition of Pragmatic Prosody in the Preschool Years and Its Relation With Theory of Mind

**DOI:** 10.3389/fpsyg.2021.662124

**Published:** 2021-07-16

**Authors:** Mariia Pronina, Iris Hübscher, Ingrid Vilà-Giménez, Pilar Prieto

**Affiliations:** ^1^Department of Translation and Language Sciences, Universitat Pompeu Fabra, Barcelona, Spain; ^2^Zurich University of Applied Sciences, Zurich, Switzerland; ^3^URPP Language and Space, University of Zurich, Zurich, Switzerland; ^4^Department of Subject-Specific Education, Universitat de Girona, Girona, Spain; ^5^Institució Catalana de Recerca i Estudis Avançats (ICREA), Barcelona, Spain

**Keywords:** prosody, acquisition, pragmatics, development, Theory of Mind (ToM), preschoolers

## Abstract

While it is well known that prosodic features are central in the conveyance of pragmatic meaning across languages, developmental research has assessed a narrow set of pragmatic functions of prosody. Research on prosodic development has focused on early infancy, with the subsequent preschool ages and beyond having received less attention. This study sets out to explore how young preschoolers develop the ability to use prosody to express pragmatic meanings while taking into account children’s Theory of Mind (ToM) development. Though ToM has been suggested to be linked to the development of receptive prosody, little is known about its relationship with expressive prosodic skills. A total of 102 3- to 4-year-old Catalan-speaking children were assessed for their pragmatic prosody skills using 35 picture-supported prompts revolving around a variety of social scenarios, as well as for their ToM skills. The responses were analyzed for prosodic appropriateness. The analyses revealed that 3- to 4-year-olds successfully produced prosody to encode basic expressive acts and unbiased speech acts such as information-seeking questions. Yet they had more trouble with complex expressive acts and biased speech acts such as the ones that convey speakers’ beliefs. Further analyses showed that ToM alone is not sufficient to explain children’s prosodic score, but the prosodic performance in some pragmatic areas (unbiased pragmatic meanings) was predicted by the interaction between ToM and age. Overall, this evidence for the acquisition of pragmatic prosody by young preschoolers demonstrates the importance of bridging the gap between prosody and pragmatics when accounting for prosodic developmental profiles, as well as taking into account the potential influence of ToM and other socio-cognitive and language skills in this development.

## Introduction

Prosody is an essential part of spoken language, and refers to suprasegmental features of speech at the word and sentence levels, such as changes in pitch, duration, and intensity. In this way, speakers typically produce utterances faster or slower, louder or quieter, and mark them with different pitch contours (intonation). Prosodic changes are well-known to encode different pragmatic meanings across languages, helping speakers to reflect intended meanings in context (Gussenhoven, 2004; Ladd, 2008). For example, a speaker can intend to focalize some information, convey uncertainty or express positive appreciation (see also examples below). Despite a growing body of literature on the prosody-pragmatics interface, relatively little of it has explored how children learn to use prosody to convey pragmatic meanings (Prieto and Esteve-Gibert, 2018; Chen et al., 2020). Although previous research has investigated early prosodic abilities in very young infants, studies on speech prosody in older children that bring prosody and pragmatics together are still rare and tend to focus on fairly specific aspects of the pragmatics-prosody interface such as the prosody of focus (Chen, 2018; Ito, 2018; see also Esteve-Gibert and Prieto, 2018, for a review) rather than providing a complete picture of children’s developmental profile. We are thus faced with a gap in our understanding of the acquisition of pragmatic uses of prosody during the preschool years and beyond. In order to address this issue, we aim here to comprehensively explore the acquisition of pragmatic prosody, that is, the ability to convey a set of pragmatic dimensions through prosody, in children aged 3–4, while taking cognitive abilities such as Theory of Mind (ToM) into account as a potential influencing factor. We next highlight the importance of considering pragmatics and prosody together, briefly review research on prosodic development, and outline what is known about the potential role of ToM in this respect.

### Prosody-Pragmatics Interface in Adult Language and in Development

It has been amply demonstrated that, across languages, prosodic cues such as intonational patterns or speech rate are central in the conveyance of pragmatic meaning (Pierrehumbert and Hirschberg, 1990). This is not surprising because prosody is never produced in isolation, dissociated from a specific pragmatic situation. Cross-linguistically, prosodic features convey a wide range of pragmatic dimensions, ranging from unbiased speech acts (for example, information-seeking questions) to biased speech acts (for example, statements encoding speakers’ beliefs) (see Prieto, 2015; Brown and Prieto, 2021, for an overview of the pragmatic meanings encoded by prosody).

For example, languages typically use distinct prosodic patterns such as falling or rising intonation to differentiate between an unbiased assertion and an unbiased request. By the same token, the information status of an element in discourse (e.g., whether it is new information or previously known) is encoded by many languages through the use of prosodic focus (see Kügler and Calhoun, 2020, for a typological review). If a person mishears, “back yard” and asks “Did you say ‘pack of cards’?,” the response will be “*No*, I said ‘*back yard*’,” with prosodic stress (in italics) expressing contrastive/corrective focus. Prosodic patterns are also involved in the marking of expressive speech acts conveying social affect; in other words, to sound socially appropriate, speakers need to produce an utterance using an appropriate tone of voice (e.g., Culpeper, 2011). For example, the appropriate prosody for the basic expressive speech act such as English greeting “Good morning” typically includes slow tempo and a wide pitch range, and the absence of these prosodic cues may have the effect of conveying indifference or rudeness. Similarly, when one is presented with a piece of freshly baked homemade pie (complex expressive acts), the comment “Mmm! It smells delicious!” will best convey positive affect if the utterance is delivered with higher pitch than usual or a temporal lengthening of the stressed syllables. Finally, epistemic states which denote knowledge and beliefs of the speaker about the propositional content of the target utterance, such as ignorance, obviousness, surprise, degree of certainty, incredulity or confirmation, can also be expressed by prosodic means across languages (e.g., Roseano et al., 2016). For example, *wh*- questions conveying surprise and curiosity, such as “*What*’s in the bag?!,” are typically realized through wider pitch excursions. In short, across languages, a broad panoply of pragmatic meanings are conveyed by means of a wide variety of prosodic and intonational strategies—what we will refer to in this paper as “pragmatic prosody.”

### An Overview of Research on Children’s Prosodic Development

Previous research on the development of prosody in children has tended to focus on early infancy (see Frota and Butler, 2018; Chen et al., 2020, for reviews). Several of these studies have investigated the essential role played by prosodic cues in very young infants for their language development. For example, it has been established that infants exploit prosodic structure to segment speech and access syntactic information (e.g., Mehler et al., 1988; Morgan and Demuth, 1996; Christophe et al., 2003; Wellmann et al., 2012; see de Carvalho et al., 2018, for a detailed review). Other studies have shown that the ability to discriminate lexical stress influences language-specific preference patterns, an ability that is important for the acquisition of language-specific prosodic properties (Bhatara et al., 2018). It has also been demonstrated that prosodic signals help infants not only with word segmentation but also with word-semantic mapping (e.g., Jusczyk et al., 1993; Jusczyk and Aslin, 1995; Friedrich and Friederici, 2004; Kooijman et al., 2013; see Teixidó et al., 2018; Thorson, 2018, for reviews). As Frota and Butler (2018) argue, these early perception abilities pave the way for the infants’ emerging abilities related to the production of intonation.

Research has demonstrated that toward the end of their first year of life infants start to master some basic pragmatic uses of prosody, such as to make requests (Esteve-Gibert and Prieto, 2018), and during the second year of life they begin to use some intonational pitch contours in an adult-like way. Specifically, in this period children’s intonational output produced in naturalistic settings tends to reflect the basic target inventory of nuclear pitch accents and boundary tones of the ambient language, and, importantly, the form-meaning relationships of some tunes are adult-like too. In a longitudinal study with four Catalan- and two Spanish-learning toddlers, Prieto et al. (2012) showed that from the first onset of speech these toddlers have a small repertoire of intonational contours that express distinct speech acts, such as requests, questions, vocatives, statements or commands (and see Frota et al., 2016, for a similar analysis involving European Portuguese-speaking children). Thus, by age 3 children are already skilled at expressing basic speech acts prosodically.

Thereafter children continue to acquire pragmatic prosody skills, albeit gradually. Different methods have been used to explore the development of prosody in the preschool and school ages, ranging from corpus-based (e.g., the CHILDES project Macwhinney, 2000) to experimental task-oriented behavioral approaches (e.g., a picture-matching task in Chen, 2009, 2011; a guessing game in Hübscher et al., 2019b). However, these methods have been applied to assess a small number of pragmatic prosody aspects. For example, elicited production experiments have examined a limited range of pragmatic uses of prosody, mostly including the prosodic contrast between assertions and requests (e.g., Patel and Grigos, 2006; Patel and Brayton, 2009) or the use of prosody to encode focus (e.g., Hornby and Hass, 1970; Chen and Höhle, 2018). From the point of view of clinical evaluation, assessment tools have also tended to include a narrow list of pragmatic functions of prosody. Such tools are primarily designed for clinical use or for research in diverse clinical populations (see Peppé, 2018) and mainly focus either on receptive prosodic skills, or on basic expressive prosodic skills. The PEPS-C (Peppé and McCann, 2003) is the only instrument that takes into account some pragmatic functions of prosody, as it assesses the production of questions and statements, the ability to place contrastive stress and the ability to express affective stances (two feelings: liking and disliking) (Wells et al., 2004, for British English; Martínez-Castilla and Peppé, 2008, for Spanish; see Filipe et al., 2017, for European Portuguese, and Filipe et al., 2018, for children with autism). To our knowledge, no previous study has comprehensively integrated the pragmatic functions of prosody through a context-based elicitation method. Below we provide a brief overview of what is known about the development of pragmatic prosody from preschool years onward, focusing on the marking of informational structure, social affect and epistemic meanings.

The ability to prosodically mark the informational structure of an utterance (i.e., focus) through intonational means has been shown to have a slow developmental trajectory in some languages. While it has been reported that children start to use intonational prominence to mark focus between 3 and 6 years of age (e.g., Hornby and Hass, 1970; Wonnacott and Watson, 2008; Romøren, 2016), typological differences between languages have been shown to affect the respective developmental trajectories for this skill (Chen, 2018). At the same time, although even preverbal children can to a certain extent both comprehend and express informational structure (Thorson and Morgan, 2014), early onset in the detection of focus does not translate into immediate mastery in production (Ito, 2018). Ito (2018) argued that the use of prosodic cues to express and comprehend focus can be affected by language-specific constraints, that is, by the language-specific repertoire of focus expressions and the availability of alternative means of focus marking such as syntactic strategies. The acquisition of focus prosody can also be affected by individual variability related to grammatical and cognitive skills, above all executive functions (see also Filipe et al., 2018, for a broader link between prosodic skills and executive functions). In fact, prior evidence of the role of executive function in various aspects of oral communication skills in both typically and atypically developing children led Ito (2018) to suggest that the growth of cognitive resources such as attention span and memory must affect, to some degree, children’s ability to map prosodic cues to informational structures. The author also warned that the methodology employed in a particular study (e.g., the experimental task performed, the type of focus under investigation, such as narrow focus vs. contrastive focus) must be carefully borne in mind when that study’s results are evaluated. This is because focus intonation is a complex skill that is in the process of development in the preschool years, and its use requires the integration of advanced semantic and pragmatic knowledge in combination with certain cognitive abilities.

Another important skill undergoing development in the preschool years (ages 3–5) is the expression and comprehension of social affect, that is, the ability to produce and comprehend basic expressive social acts such as greeting or thanking, as well as more complex expressive social acts such as congratulating or voicing concern. As children grow up and as the social world around them becomes more complex, emotional and social competencies become closely interconnected (Denham et al., 2011). By preschool, children’s social tasks include identifying a speaker’s emotion and being able to convey their own emotions appropriately in keeping with the ongoing context. Though recent research has pointed to the importance of prosodic cues in signaling and inferring social meanings such as politeness and assessed its development in 3- to 5-year-old children (Hübscher et al., 2019a, 2020), relatively little attention has been paid to the prosodic strategies employed by preschoolers in social interactions to perform expressive social acts, whether basic or complex.

Similarly, the ability to prosodically express epistemic states such as uncertainty, disbelief, obviousness or surprise develops over a long period and full adult-like competence is achieved only gradually. Awareness of a speaker’s epistemic state (e.g., a knowledgeable vs. an ignorant speaker) based on contextual evidence (not prosodic cues) is present even in 12-month-old infants (Liszkowski et al., 2008). However, various cross-sectional studies have demonstrated that it is in the time window between 3 and 5 years of age that children start to employ prosodic signals to comprehend and express epistemic meanings (Hübscher et al., 2017, 2019b; Armstrong, 2018; Armstrong et al., 2018). In a study investigating the expression of (dis)belief in 1- to 3-year-olds, Armstrong (2018) found that around 3 years of age the children began to be able to express their belief about propositional content through polar questions (e.g., they could convey a belief that there was going to be a party the next day by asking a question like “Is there a party tomorrow?”). However, the children were not able to express disbelief, incredulity or doubt about the propositional content (e.g., they could not produce the counter-expectational question “There’s a party tomorrow?!”).

The ability to use prosodic cues to comprehend and convey epistemic states continues to develop over the preschool period (Hübscher et al., 2017; Armstrong et al., 2018). For example, 4- and 5-year-old children are better at comprehending disbelief through prosody than 3-year-old children (Armstrong et al., 2018). As for production, likewise, children start to signal the epistemic meaning of uncertainty through prosodic and gestural markers between the ages of 3 and 5, and this ability develops as they get older (Hübscher et al., 2019b). Interestingly, young preschool children do not use prosody to express epistemic meanings in exactly the same way as adults do. For instance, 3- to 4-year-olds are able to use rising pitch contours expressing uncertainty, but other strategies typically employed by adults (e.g., fillers and vowel lengthening) only begin to emerge later, starting around age 5 (Hübscher et al., 2019b). Armstrong (2018) tentatively proposed to explain her findings about the late developmental window for the acquisition of epistemic markers in terms of cognitive developmental constraints. That is, in order to be able to utter a disbelief contour the child must be able to hold in mind simultaneously a previous belief and new information that has become available in the discourse in order to compare them. In this view, non-felicitous uses by children of contours expressing speaker disbelief can be attributed to their immature conceptual understanding of epistemic states.

### Relationship Between Prosodic Development and Theory of Mind

As proposed in a recent overview on the development of mental state prosody by Armstrong and Hübscher (2018), the acquisition of epistemic intonation could be related to factors such as belief understanding. In order to comprehend epistemic states expressed through prosody, the child must have developed the capacity to understand the mental states of others, known as ToM (Premack and Woodruff, 1978). Children’s ToM understanding is a crucial cognitive competence that develops gradually during childhood and undergoes great changes during the preschool period (Wellman, 2018). Research investigating ToM abilities in preschoolers has mostly focused on one key aspect of ToM, namely, explicit false-belief understanding (see Wellman and Liu, 2004, for a review). The test most frequently used to assess ToM development in this period is the so-called false belief task which focuses on a child’s ability to predict the actions of a person holding a mistaken (false) belief. Since it is not until around the age of 4 that typically developing children succeed in false-belief tasks (Sodian, 2006; Wellman, 2018), its use is ideal for the present investigation involving young preschoolers (i.e., 3- to 4-year-olds).

With respect to the relationship between the acquisition of epistemic meanings and ToM, previous research has suggested that ToM understanding can be linked to epistemic vocabulary (see De Rosnay and Hughes, 2006; Slaughter and Peterson, 2012; Ebert et al., 2017, for the acquisition of mental state lexicon) and morphosyntax (e.g., see Matsui et al., 2009 for the acquisition of grammaticalized means for evidential and certainty marking in Japanese). However, studies on the relationship with epistemic prosody are few in number (e.g., Armstrong et al., 2018; Esteve-Gibert et al., 2020). Empirical evidence for such a relationship comes from the study on the comprehension of disbelief conducted by Armstrong et al. (2018). The results revealed that the ability to perceive disbelief through intonation in 3- to 5-year-old children was predicted by the stage they had reached in the development of their ToM. To our knowledge Armstrong et al.’s (2018) experiment is the only one that includes ToM measures in relation to the development of epistemic prosody. One study involving adult participants found that empathy skills (sometimes understood as ‘affective’ ToM, see Harwood and Farrar, 2006; Hughes et al., 2007, for details) are linked to individual variation in receptive prosodic skills (Esteve-Gibert et al., 2020). Specifically, it was shown that more empathetic individuals are more sensitive to intonational cues, while less empathetic individuals have trouble disambiguating a speaker’s intentions on the basis of their intonation.

However, some contradictory evidence for the relationship between prosody and ToM comes from studies on atypical development. For instance, some studies have failed to detect any relationship between receptive prosodic skills and ToM (Chevallier et al., 2011; Colich et al., 2012). For example, Chevallier et al. (2011) found that children with autism spectrum disorder (ASD) were as good as typically developing children in processing prosody, suggesting that impaired ToM does not entail difficulty in reading other people’s minds from vocal cues. As for expressive prosody, it is widely known that individuals with ASD and other disabilities often present unusual prosody (see McCann and Peppé, 2003; Loveall et al., 2021, for a detailed review) but research still is lacking on the association between prosodic impairments and ToM. Summarizing, past research on the relationship between children’s prosody and ToM is admittedly limited and has mainly focused on epistemic prosody and receptive prosodic skills, which suggests that more research should be undertaken to assess this complex issue.

### Main Goals and Hypotheses

All in all, the state of the art on prosodic development reveals a rather fragmentary picture of the development of pragmatic prosody, with studies mainly focusing only on the early stages of the detection or comprehension of pragmatic meanings conveyed by prosodic means, with much less being known about the subsequent stages of development between ages 3 and 4. The main aim of the present study is thus to explore the development of pragmatic prosody skills in typically developing young preschool children in that period. The range between 3 and 4 years of age was selected as a focus for this study for several reasons. First, previous research has demonstrated that children’s production of epistemic- and politeness-related meanings starts in the early preschool age, thus, this age range is especially relevant with regard to different pragmatic competences (Hübscher et al., 2019a, b). Second, interestingly, numerous studies have consistently shown that critical development of ToM takes place in this period (Wellman, 2018).

Crucially, as noted above, prior research has tended to focus on specific pragmatic functions of prosody, such as the conveyance of information focus and belief states. Here we will attempt to take a more holistic, comprehensive measure of children’s communicative uses of prosody by using the Audiovisual Pragmatic Test (APT; Pronina et al., 2019). The APT has been specifically designed to track the acquisition of pragmatic prosody skills in children starting from the preschool age. We hypothesize that the route taken in the acquisition of pragmatic prosody will vary depending on the nature of the specific pragmatic area, with the prosody of unbiased and basic speech acts being acquired first and the prosody of biased pragmatic dimensions such as information focus and epistemic state being acquired later and to a lesser degree in this period.

Finally, a further goal of the study is to assess whether the acquisition of pragmatic prosody skills in children is linked in any way with their development of ToM abilities. Here we hypothesize that, in line with previous research that suggested the link between ToM and receptive prosodic skills in children and adults (e.g., Armstrong et al., 2018; Esteve-Gibert et al., 2020), ToM will be related to expressive prosodic skills across various pragmatic areas.

## Materials and Methods

### Participants

A total of 117 3- to 4-year-old children were initially enrolled in this study, all of them preschoolers at two Catalan public schools located in the middle-income district of Sant Martí within the metropolitan area of Barcelona, where the population is largely Catalan-Spanish bilingual and the main language of instruction is Catalan. Prior to the experiment, the children’s caregivers signed a participation consent form and filled out a language questionnaire (based on Bosch and Sebastián-Gallés, 2001) regarding the daily exposure of their child to Catalan. They also completed an occupational status questionnaire that was used to assess socio-economic status (SES). Caregivers’ responses on their occupation were coded according to the International Socio-Economic Index (ISEI; Ganzeboom et al., 1992), a continuous occupational index that categorizes occupations into job categories, with higher job category score indicating higher occupation status. According to caregivers’ reports, all enrolled participants were typically developing children and had no history of speech, language or hearing difficulties.

Prior to initiation of the study proper, participating children were given a Catalan language test (Saborit Mallol et al., 2005) that is specially designed to evaluate expressive vocabulary skills in children aged 2 to 9. This was done to ensure that participants’ command of Catalan would be sufficient to allow them to perform the tasks they would be asked to do. The mean vocabulary score for the 117 participants was 31.23 out of 100 (*SD* = 12.77, ranging from 0 to 70). A score of 20 was set as the eligibility threshold for participation. Of the 117 children who had been initially enrolled in the study, 15 failed to reach this threshold. This left a final study population of 102 children (45 males, 57 females), with ages ranging from 39 to 51 months (*M*_*age*_ = 44.92 months, *SD* = 3.29 months). The mean overall Catalan exposure time of these children was 57% (*SD* = 23). The children came from middle socioeconomic status families (*M*_*SES*_ = 61.16, *SD* = 12.73).

### Materials

#### Theory of Mind (ToM)

Theory of Mind was assessed using two classic false belief tasks that measure a child’s ability to understand others’ mental states. The first one was Gopnik and Astington’s (1988) unexpected content task. In this task, each child was shown a plastic tube, the usual packaging for *Lacasitos* colored chocolate disks (the local analog to the Smarties tube used in the original task described in Gopnik and Astington, 1988), and was then asked what it contained. This consistently produced the expected answer “Caramels” (“Candies”). The tube was then opened to reveal that it contained bits of chalk. After seeing these contents, the child was asked what they had thought was in the box before it was opened and what a friend of theirs would think was inside the box before it was opened.

The second was a version of the unexpected location task described in Baron-Cohen et al. (1985) adapted to Catalan (Armstrong et al., 2018). In this task, each child was shown a video in which a princess puts a ball in a container and leaves the scene. A lion then appears, moves the ball from the container where the princess left it to a second container, and leaves the scene. Finally, the princess returns to the scene. At this point, the child was asked where the princess would look for the ball and where the ball really was.

#### Audiovisual Pragmatic Test (APT)

The Audiovisual Pragmatic Test (APT) was developed to jointly test pragmatic and prosodic abilities from early childhood, starting from the age of 3, until late childhood in typically developing children (see Pronina et al., 2019, for a detailed explanation of the task; materials are available from the Open Science Framework online repository^1^). First, its pragmatic coverage is appropriate for children starting from age three as it takes into account widely used standardized pragmatic tests designed to assess communicative behavior in children such as the *Test of Pragmatic Language* (TOPL-2; Phelps-Terasaki and Phelps-Gunn, 2007) and the *Comprehensive Assessment of Spoken Language–2* tool (CASL–2; Carrow-Woolfolk, 2017). Second, it uses a carefully controlled picture-supported set of contextual prompts, allowing the user to assess children’s prosody in relation to pragmatic social contexts (i.e., pragmatic prosody). The elicitation procedure follows the Discourse Completion Task methodology, in which the participants are asked to imagine a pragmatic scenario and then to respond to it using their own words. This procedure has been widely used to research both pragmatics and prosody and has been proven to be an effective, reliable and validated method in the field of prosody (Vanrell et al., 2018). The procedure is suitable for children as the everyday situations presented in the items are adapted for a child’s everyday life and are presented to children on a role-play basis, which allows them to immerse in the social situations. Moreover, all items are supported with colored pictures, which help children to imagine the situations and minimize memory load. Children are asked to respond as naturally as possible and can freely utter any response. The APT was tested with 3- to 8-year-old children and it was found that children of all ages engage in the activity (42% of 3- to 4-year-olds, 94% of 5- to 6-year-olds and 100% of 7- to 8-year-olds respond all the items) and are able to produce (semi-) spontaneous speech in response to the APT items (Pronina et al., in preparation).

The full version of the APT includes 47 items which depict a specific pragmatic situation. Test administration involves the examiner picturing the social situation in a lively child-directed fashion while the child looks at the illustration displayed on the computer screen. The examiner asks the child to respond appropriately as if (s)he was an actual participant in the situation. If the child shows any difficulty understanding a situation or does not behave as expected, the examiner tries to clarify the situation further by replacing the fictional characters in the prompt with the names of people who were likely to be important to the child (such as a friend, parent or teacher). For example, if the child seems to have trouble imagining a friend offering to share half of his muffin, the experimenter would ask the child to name one of their friends in real life and then would frame the situation as if that friend was the main character in the social sharing situation.

Each item is intended to elicit a pragmatically appropriate verbal response which corresponds to one of four speech acts, namely *assertions*, *requests*, *basic expressive acts* and *complex expressive acts*, with assertions and requests being either *unbiased* or *biased*. Unbiased requests and assertions have no additional pragmatic meanings. An unbiased assertion has a declarative or explanation illocutionary force and no markers of modality, as exemplified by an unmarked declarative statement (example 1 in Table 1). An example of an unbiased request would be a command (example 2), a neutral information-seeking question (example 3), or a request for permission (example 4). Biased requests and assertions convey additional pragmatic biases that add complementary meanings to them (see Krifka, 2015, 2017, 2019). In line with Krifka’s account, we differentiate between negation and epistemic biases, but we also add focus bias. For example, a request or an assertion can have marked informational structure, that is, focus (example 5), express different types of epistemic meanings (example 6) or negation (example 7). *Basic expressive acts* correspond to basic social acts such as greeting, bidding farewells, thanking or apologizing (examples 8 and 9; see Norrick, 1978). *Complex expressive acts* revolve around complex social situations like expressing compassion, condolences, congratulations or praise toward a peer, a parent or a teacher (examples 10 and 11). These items typically require the child to produce a positive exclamation or to communicate a positive stance conveying appreciation of or emotional support for the person to whom they are speaking.

**TABLE 1:** Speech acts tested by the APT, with sample prompt context descriptions and corresponding illustrations.

	Speech act	Pragmatic biases	Context description (read by experimenter to child)	Illustration (viewed by child)
1	Assertion	Unbiased	*Imagine that you’re eating a piece of cake and when you finish, your aunt asks you, “Do you want more?” What would you say?*	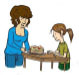
2	Request	Unbiased	*Imagine that you’re in a park with your family and your parents ask you to look after your little sister. But suddenly she runs out of the park. You’re worried because there’s a lot of traffic and you’re afraid she’ll step into the street. What would you tell her?*	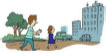
3	Request	Unbiased	*Imagine that there is there’s a new girl in your grade at school. You like music class very much. One day you start talking with her and you want to know if she’s also taking music class. How would you ask her?*	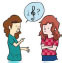
4	Request	Unbiased	*Imagine that you want to watch TV and you know that usually your parents don’t allow you to. How would you ask permission from your parents?*	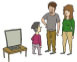
5	Assertion	Biased (focus)	*Imagine that you are in your grandmother’s house and she can’t hear well. You just told her that you want a snack because you are hungry but she did not hear you and asks, “Do you want to go for a walk?” How would you tell her that that’s not what you want, you want a snack instead?*	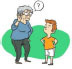
6	Request	Biased (epistemic bias of surprise)	*Imagine that 1 day your mother comes home carrying a very big bag. You’re very curious about what’s in the bag. What would you say to your mother?*	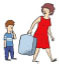
7	Assertion	Biased (negation)	*Imagine that you do not like bananas but your mother gives you one. She is very sure you like them. You want to tell her that you do not like bananas. What would say?*	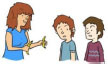
8	Basic expressive act		*Imagine that you walked into your classroom in the morning. What would you say to your teacher?*	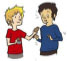
9	Basic expressive act		*Imagine that you just entered the classroom in the morning. What would you say to your teacher?*	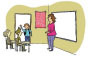
10	Complex expressive act		*Imagine that you come home and when you enter the kitchen you see your mother baking and smell a delicious pie. What would you tell her?*	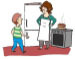
11	Complex expressive act		*Your friend just tripped and fell down. What would you say?*	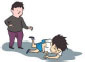

For the purposes of the study, given that the test-takers were 3- to 4-year-old children, only the first 35 items out of a total of 47 were used. We predicted that the last 12 items would not be appropriate for this age range since they were tied to social contexts that preschool children would not be likely to encounter (for example, having to politely refuse to give personal information).

### Procedure

During their regular class time, the children were accompanied individually to a quiet room at their respective preschools by the examiner (the first author) or one of three trained research assistants, and then underwent the assessment procedures. Assessment took place in two separate sessions of a total duration of 30 min, though each child was supervised by the same experimenter in both sessions.^2^ In the first session the child performed the two ToM assessment tasks while in the second session the APT was carried out. All four experimenters were fluent Catalan speakers and all testing was conducted in Catalan. The duration of the APT was between 15 and 20 min (this variability is explained below). The total duration of the two ToM tasks together was approximately 5 min, all participants were able to provide answers to the questions and complete both ToM tasks. All assessment sessions were video-recorded.

For the APT, the child was asked to sit in front of the computer screen where the pictures illustrated the social situation serving as prompts would be presented. The experimenter first engaged in a warm-up conversation with the child, took them through two sample items to make them familiar with the test procedure and then proceeded with the test itself. Although the version of the APT used here consisted of 35 items, the test was stopped before if the child showed signs of lack of collaboration or fatigue. The experimenter could interrupt the session for little breaks. However, the test was stopped if the child was unwilling to continue. For example, if the child answered “I don’t know” or remained silent over several items, the test was discontinued. This procedure is in line with the guidelines of similar developmental batteries (e.g., CASL-2, CELF-2 Preschool). In total, this occurred in the case of 59 out of the 102 participating children. Two children explicitly asked to stop the task or return to his/her class; 36 children ceased to collaborate over several items, either by saying “I don’t know” in response to the prompts, providing inappropriate responses like “Please” or “Yes” to all prompts, or failed to respond verbally at all; 15 children became too restless to remain on task; and 6 children became excessively distracted and/or tired.

### Scoring

Before testing, all experimenters were trained for scoring of the ToM tasks and the APT in a 1-h session. Then they carried out the scoring online while administering the tests using previously prepared answer sheets. Scores were then carefully checked by the first author on the basis of the session video-recordings.

#### Theory of Mind (ToM)

As described above, in the unexpected content task, each child was asked what they originally thought was in the Lacasitos tube, and if they answered “Candies” they were awarded one point. If, when asked what a friend of theirs would think was in the tube, they answered “Candies,” they were awarded a second point, for a possible total of two on this task. In the unexpected location task, each child was asked where the princess would think the ball was and the ball actually was. If they answered “In the first container” to the first question they received one point, and if they answered “In the second container” to the second question they received a second point, for a possible total of two. Thus the total ToM score ranged from 0 to 4.

#### Audiovisual Pragmatic Test

For the APT, two complementary scores were given, one for pragmatic appropriateness and the other for prosodic appropriateness. The pragmatic score was used only for screening purposes, since pragmatically inappropriate responses were excluded from the prosodic analysis.

##### Pragmatic appropriateness score

The pragmatic component of each response was given a score from 0 to 2. The scores were based on the examiner’s perception of whether a given response expressed the intended speech act and was socially and contextually appropriate. A score of 2 was recorded if the child’s answer was of high pragmatic quality, that is, the child managed to produce the intended speech act and did so in a socially appropriate way, such as if when asking for a piece of cake, the child used a polite question like ‘‘Can I have a piece?’’ A score of 1 was given if the child managed to utter the expected speech act but showed a lack of social appropriateness, which usually meant, depending on the scenario that the child either said too little or too much or answered too directly. For example, in the context of asking for a cake, if the child bluntly said ‘‘Give me’’ or ‘‘I want cake’’ this response was scored as 1 since the child was able to express the request but failed to mitigate it politely as would be appropriate for the situation. A score of 0 was given if the child either produced a response that did not match the relevant speech act or failed to provide any response whatsoever^3^. In the context of asking for more cake, for example, a response like “I like cake” would receive a score of 0.

##### Prosodic appropriateness score

As noted, the prosodic component of a child’s response was only assessed if it had first received a score of 1 or 2 for pragmatic appropriateness. Prosodic appropriateness was scored in a similar fashion, that is, by perceptually assessing the prosodic felicitousness of the response relative to the particular context. Given the fact that specific pitch contours and prosodic patterns encode a set of pragmatic meanings, in the assessment of prosodic felicitousness, we did not carry out an intonational analysis of pitch contours and prosody was assessed holistically by simultaneously considering the effect that different dimensions of prosody (e.g., intonation, amplitude and duration) produce on the listener. This is in line with previous clinical and developmental research (Rasinski, 2004; Papakyritsis, 2021).

**FIGURE 1:**
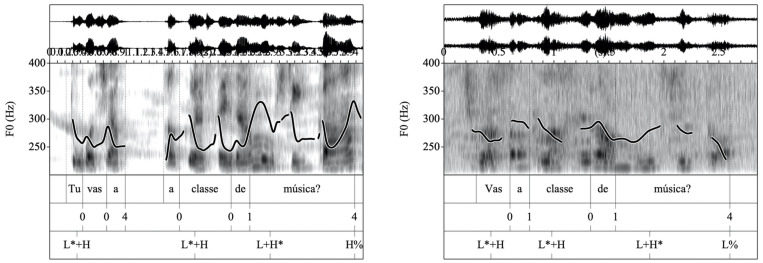
**(Left)** Waveform, spectrogram and F0 trace for the question “*Tu vas a classe de música?*” “Are you taking music class?” scored 2 for prosody and produced with two prenuclear L* + H pitch accents and a L + H*H% nuclear configuration. **(Right)** Waveform, spectrogram and F0 trace for the same question, this time scored 1 for prosody and produced with two prenuclear L* + H pitch accents and a L + H*L% nuclear configuration, which is typical for statements.

**FIGURE 2:**
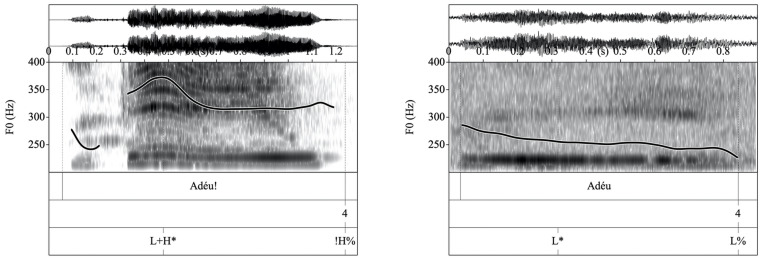
**(Left)** Waveform, spectrogram and F0 trace for the prosodically appropriate expressive farewell “Adéu!” “Bye!” scored 2 for prosody and produced with a L + H* nuclear configuration and followed by a !H% boundary tone. **(Right)** Waveform, spectrogram and F0 trace for the same utterance with inappropriately unexpressive prosody in the form of a L* nuclear accent and followed by a L% boundary tone. This utterance would receive a score for prosodic appropriateness of 1.

The prosodic component of the response was perceptually assessed as felicitous prosody, infelicitous prosody, or indirect speech. Prosody was scored as felicitous if the child used direct speech in first-person and answered with the prosody that would be appropriate if the situation was really happening at that moment. Because various prosodic strategies may be employed by a speaker to encode a certain particular pragmatic meaning, no single prosodic pattern was regarded as uniquely appropriate for an item, and the prosodic means used by the child were evaluated in terms of perceived felicitousness in each particular instance. For example, consider a context in which the child was prompted to ask a peer whether she was also taking music class. If the child answered “Are you taking music class?” with the corresponding interrogative intonation contour (Figure 1, left), prosody was scored as felicitous. If in another context the child was prompted to produce an affectionate farewell to the mother and (s)he said “Bye!” with a naturally sounding rising-falling pitch contour, the answer was also scored as felicitous (Figure 2, left).

In contrast, prosody was scored as infelicitous if there was a mismatch between the pragmatic meaning that should have been communicated by the response and the prosody used. For example, if in asking whether a peer was also taking music class the child produced a pragmatically appropriate response such as “Are you taking music class?” but the intonational contour was not interrogative but assertive (see Figure 1, right), infelicitous prosody was recorded. Prosody was also scored as infelicitous if the child’s response conveyed very low prosodic expressiveness. These answers were typically characterized by very narrow pitch range variation or reduced intensity and therefore sounded unnatural. For example, if the child was prompted to produce an affectionate farewell to his/her mother, and the child said “Bye” using non-expressive flat intonation, the answer was scored as infelicitous (see Figure 2, right), as it was deemed not to correspond with the target pragmatic meaning. It is important to note that a lack of expressiveness did not render the answer non-appropriate: the child still managed to produce a pragmatically appropriate response.

Finally, prosody was scored as indirect speech if the answer was pragmatically appropriate but the child did not enact the scenario; that is, if (s)he did not take the perspective of the situation’s character. For example, if, to the experimenter’s question “What would you say?” in the situation where a piece of cake is offered, the child answered “That I want some cake” or “I would say that I want some cake,” using indirect speech to describe his/her actions instead of enacting the direct response, the pragmatic prosody of the answer cannot be evaluated as the target sentence is embedded. It should be noted that the answers produced in indirect speech cannot be classified into prosodically felicitous or infelicitous for the provided pragmatic situation, and thus are rendered as non-eligible for the prosodic scoring.

At this point, it is important to highlight that pragmatic and prosodic components of the answer were assessed separately. The reason behind this separation is because we do not wish to penalize children’s prosodic scores on the basis of pragmatic adequacy. Thus, an answer deemed as highly pragmatically appropriate (a score of 2) could be scored either as prosodically felicitous, infelicitous, or indirect, depending on the prosody used by the child. Likewise, an answer that lacks some pragmatic adjustment (a score of 1, e.g., a request produced as an imperative) could be scored either as prosodically felicitous, infelicitous or use of indirect speech. In this way, a child’s prosody score is not penalized based on the pragmatics score.

##### Inter-rater reliability

The APT results were double checked and rescored if needed by the first author of this study. The reliability of her scoring on the APT was confirmed by checking her scoring of a subset of the APT results from this study (910 responses representing 26 participants, 25% of the collected data) against scores awarded to the same items by two independent, previously trained scorers, each working separately. Using the R package *irr*, version 0.84 (Gamer et al., 2012), the scores awarded to these responses by each rater were checked against the scores awarded by each of the other two raters to determine the inter-rater reliability. Since more than two raters were involved, the inter-rater agreement was measured using Fleiss multi-rater kappa. According to Fleiss et al. (1981) benchmarks for interpreting the values of kappa, results ranging from 0.75 to 1.00 are considered “excellent” and results between 0.40 and 0.75 indicate “good agreement beyond chance.” Overall agreement between the two independent raters and the original coder was 83% for pragmatic scores and 87% for prosodic scores. Fleiss Kappa was 0.79 (*p* < 0.001) and 0.81 (*p* < 0.001) for pragmatic scores and prosodic scores correspondingly. These results suggest a high degree of inter-rater reliability among raters for both pragmatic and prosodic scores.

### Data Analyses

Two sets of analyses were performed on this dataset, all of them using R, release 4.0.2 (R Core Team, 2020). All missing data was excluded from the analysis; that is, we excluded all missing answers to the items that were not administered to the children due to the discontinuation of the APT (see also section “Procedure” for details for the interruption of testing), as they do not provide any information about a child’s prosodic abilities. Thus, we focus only on the items that were presented to the children.

Our first analysis was limited to the data obtained by means of the APT (Analysis 1, section “Analysis 1. Pragmatic Prosody”). The goal was to assess the role of speech act type on the production of pragmatically and prosodically appropriate cues. For this purpose, the 35 APT items were grouped into one of six broad categories of speech act type (namely, unbiased assertions, biased assertions, unbiased requests, biased requests, basic expressive acts, complex expressive acts). Linear mixed-effect models using the *lme4* software package, version 1.1-23 (Bates et al., 2015) were run to compare children’s performance on different speech act categories. A first linear mixed-effect model tested whether there was a significant difference in the children’s prosodic performance on unbiased and biased speech acts. We fit the model for the data on unbiased assertion, biased assertions, unbiased requests and biased requests. Prosodic Score (felicitous vs. infelicitous vs. indirect) was set as the dependent variable; Bias (unbiased vs. biased speech act) was set as predictor; Participant and Item were entered as random factors. In the second linear mixed-effect model, we tested whether the difference between the children’s prosodic performance on basic and complex expressive speech acts was significant. This model was fitted for the data on the basic and complex expressive acts with Prosodic Score (felicitous vs. infelicitous vs. indirect) as the dependent variable, Speech Act (basic vs. complex expressive speech act) as the fixed effect and Participant and Item as random factors. We further run additional models that compared the prosodic performance in (a) different types of biases and (b) different types of epistemic biases. Again, Prosodic Score was entered as dependent variable and Participant and Item were set as random factors, but in one model Bias Type was used as independent variable (unbiased vs. negation vs. focus vs. epistemicity) and in another one Epistemic Bias (surprise vs. confirmation vs. incredulity vs. obviousness vs. uncertainty) was set to be independent variable.

Second, we analyzed the relationship between pragmatic prosody, ToM and age in months (Analysis 2, section “Analysis 2. Relationship Between Pragmatic Prosody, ToM and Age”). Here, we conducted a series of linear mixed-effect models, also using the *lme4* software package, version 1.1-23 (Bates et al., 2015). The first model included all pragmatic prosody areas. Prosodic Score (felicitous vs. infelicitous vs. indirect) was set as the dependent variable. Nested models were compared using Akaike’s information criterion (AIC), and the best-fitting model included both age in months, ToM and their interaction as fixed effects. Participant and Item were set as random factors. The predictors were centered at their mean before they were entered in the analysis. Finally, a series of linear mixed-effect models were run for different speech acts (i.e., expressive, unbiased and biased speech acts). The same model specification as in the first model (i.e., Prosodic Score as the dependent variable, age in months, ToM and their interaction as fixed effects, Participant and Item as random factors) was used in all the models that were conducted for speech acts.

Let us keep in mind that though the original APT consists of 47 items, only 35 were used in this study (as the remaining 12 items described complex social situations that preschoolers would be unlikely to have faced in real life). After administration of the APT, this was found to also be true of one of the items that had been included, as indicated by the fact that none of our participants proved able to understand the context or provide a pragmatically felicitous response to it. Therefore, our analyses are based on data taken from only 34 APT items.

## Results

### Analysis 1. Pragmatic Prosody

Our intention was to use the results of the first analysis to build a kind of collective profile for the pragmatic prosody skills of this population (i.e., Catalan-speaking children aged 3 to 4). Our analyses centered around the set of speech acts which this age group is able to successfully perform using prosody. As explained, the prosodic component of the APT answers was assessed perceptually. This is consistent with previous research (e.g., perceptual assessment is considered a gold standard in clinical contexts, Rasinski, 2004; Papakyritsis, 2021, for perceptual assessment of speech prosody in retelling and reading tasks).

**FIGURE 3:**
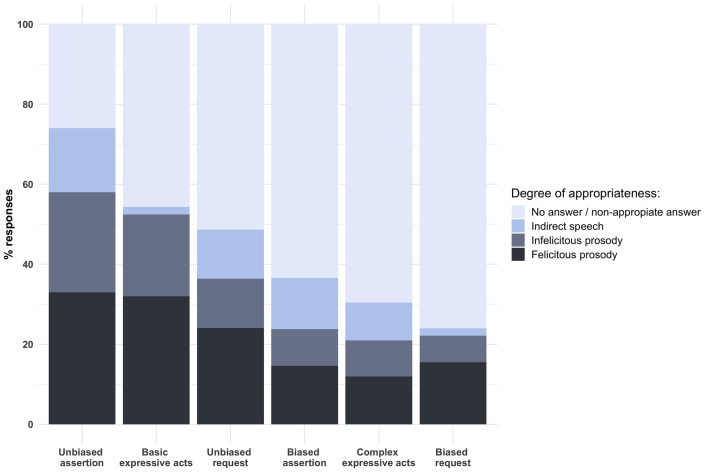
Percentage of prosodically felicitous responses by children, broken down by speech act category and degree of appropriateness.

As noted in Section “Audiovisual Pragmatic Test (APT),” the 34 items tested in the APT each fell within one of six broad categories of speech act, namely unbiased assertions, biased assertions, unbiased requests, biased requests, basic expressive acts and complex expressive acts. Also recall that the APT prosodic appropriateness scoring system distinguishes between three degrees of appropriateness, as follows: lowest degree—pragmatically felicitous answers produced as indirect speech (i.e., the child was not able to answer from the perspective of the character, that is, was not able to enact the answer); intermediate degree—pragmatically felicitous answers enacted but prosodically infelicitous (e.g., non-expressive or produced with non-adequate prosody); and highest degree—answers that are felicitous both pragmatically and prosodically. The responses by 102 children were then broken down by speech act category and prosodic appropriateness level. This was then converted to a percentage by dividing each total by the total number of items within the speech act category administered to the children (missing answers were excluded). The results are shown in Figure 3.

In this figure, speech act categories are ordered from left to right according to their success in retrieving appropriate responses (and appropriate prosodic responses) from children. Overall, it can be seen that preschoolers are able to produce appropriate responses to all speech act categories, although there are clear differences in the route of their acquisition. While a majority of children managed to successfully produce appropriate unbiased assertions, basic expressive acts and unbiased requests—albeit not necessarily with expressive prosody—biased speech acts, either assertions or requests, and complex expressive acts proved more difficult. By the same token, the highest proportions of prosodically felicitous answers are concentrated around APT items eliciting unbiased assertions (33%), basic expressive acts (32%) and unbiased requests (24%), while APT items eliciting biased assertions, complex expressive acts, and biased requests yielded prosodically felicitous responses 16% of the time at most.

These results suggest that biased pragmatic meanings are more challenging than unbiased meanings for young preschoolers. This difference was proven to be statistically significant. The first linear mixed-effect model showed that the preschoolers performed significantly better on unbiased pragmatic meanings items than on biased pragmatic meaning items (β = −0.358, *t* = −2.820, *p* = 0.010). The second linear mixed-effect model showed that preschoolers also performed significantly better on basic expressive acts compared to complex expressive acts (β = −0.559, *t* = −3.389, *p* = 0.007). The full estimates are given in Table 2.

**TABLE 2:** Model specification and estimates for the models exploring the differences in the prosodic performance for different speech act types.

Fixed effects	β	*SE*	*t*	*p*
***Pragmatic bias (biased items vs. unbiased items)***				

Intercept	0.63	0.10	6.459	<0.001***
Bias	−0.36	0.12	−2.820	0.010**

***Expressive speech acts (basic expressive vs. complex expressive)***				

Intercept	0.83	0.12	6.811	<0.001***
Expressive speech act type	−0.56	0.17	−3.389	0.007**

**p < 0.010, and 0.007, ***p < 0.001.

**FIGURE 4:**
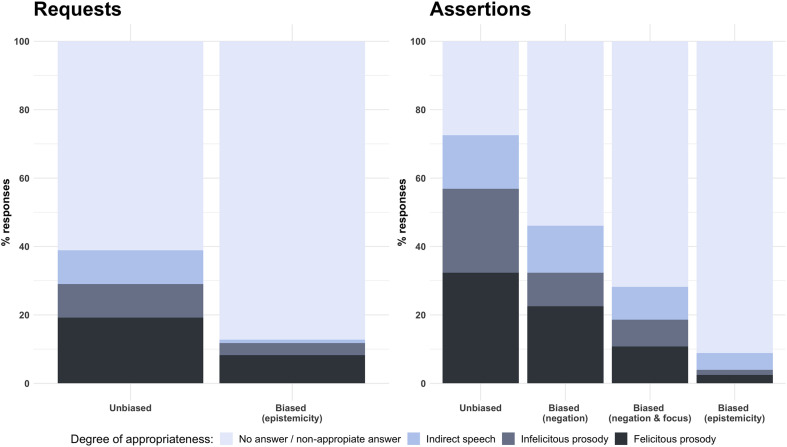
Percentage of prosodically felicitous responses to unbiased versus biased requests and assertions.

The same data is shown in Figure 4, though there the results for expressive acts are excluded and biased assertions are further broken by type of bias. This allows us to see that preschoolers seem to be more successful at conveying some degree of negative bias (51%) relative to other types of bias, most notably epistemic bias (12%). Additionally, we broke this effect down by conducting contrast analyses between bias types. The only contrast that achieved significance level was the contrast between unbiased speech acts and speech acts conveying epistemic biases (β = 0.629, *t* = 6.532, *p* = 0.006).

**FIGURE 5:**
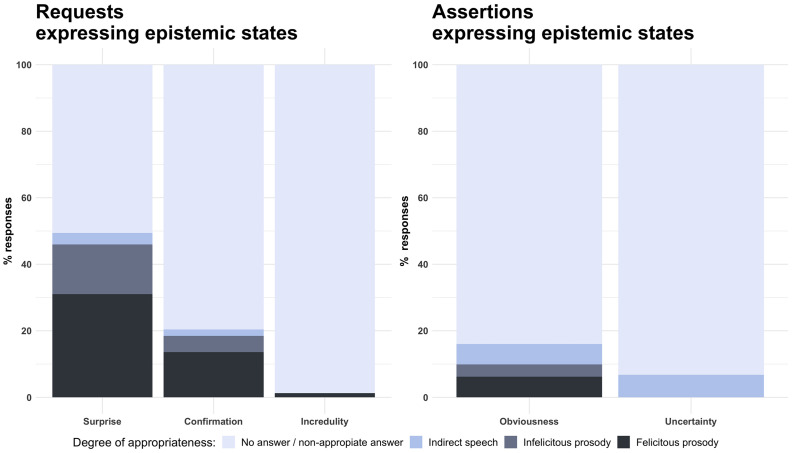
Percentage of prosodically felicitous responses by children for requests and assertions expressing epistemic stances.

Figure 5 focuses more narrowly on the data related to biased epistemic meanings. Here we find clear differences between the children’s ability to convey epistemic stance in request and assertion speech acts. While participants were able to express surprise about an object of interest (49%) or utter a confirmation-seeking request (21%), they tended to struggle with other epistemic meanings such as obviousness (16%), uncertainty (7%) and incredulity (1%). The contrast between surprise bias and uncertainty bias turned out to be significant (β = 0.770, *t* = 10.784, *p* ≤ 0.001).

### Analysis 2. Relationship Between Pragmatic Prosody, ToM and Age

Since previous research has suggested that the acquisition of prosody is related to ToM development, in this section we report on the results of the linear mixed-effect models that were intended to assess the relevance of ToM on the children’s overall prosodic performance, on the one hand, and their prosodic performance for specific speech act categories, on the other.

The first linear mixed-effect model was fit for the Prosodic Score involving all pragmatic areas (i.e., all items were entered in the model) and showed that Age, ToM and their interaction were not significant predictors of the Prosodic Score. Table 3 shows the full estimates for the fixed effects.

**TABLE 3:** Model specification and estimates for the models exploring the relationship between ToM and prosodic abilities (children’s overall prosodic performance and their prosodic performance for specific speech act categories).

Fixed effects	β	*SE*	*t*	*p*
***Overall prosodic performance***				

Intercept	0.44	0.07	6.302	<0.001***
Age	0.06	0.04	1.747	0.084
ToM	0.05	0.04	1.225	0.224
Age: ToM	0.06	0.03	1.865	0.065

***Basic expressive acts***				

Intercept	0.81	0.15	5.539	0.001**
Age	0.12	0.05	2.243	0.027*
ToM	0.01	0.06	0.093	0.926
Age: ToM	0.03	0.05	0.631	0.530

***Unbiased meanings***				

Intercept	0.57	0.09	6.598	<0.001***
Age	0.06	0.05	1.236	0.219
ToM	0.03	0.05	0.474	0.637
Age: ToM	0.13	0.04	2.917	0.004**

***Complex expressive acts***				

Intercept	0.26	0.09	2.770	0.032*
Age	0.04	0.04	0.906	0.367
ToM	0.07	0.04	1.664	0.100
Age: ToM	0.07	0.04	1.840	0.069

***Biased meanings (epistemic)***				

Intercept	0.25	0.14	1.808	0.118
Age	0.04	0.04	1.137	0.259
ToM	0.03	0.04	0.870	0.387
Age: ToM	−0.03	0.03	−0.873	0.385

***Biased meanings (focus)***				

Intercept	0.40	0.15	2.665	0.069
Age	0.00	0.06	0.033	0.974
ToM	0.10	0.06	1.675	0.099
Age: ToM	0.08	0.05	1.608	0.113

*p < 0.05, **p < 0.01, ***p < 0.001.

Given that previous research has highlighted the role of ToM for the acquisition of prosodic patterns expressing epistemic states (Armstrong and Hübscher, 2018), we carried out a set of analyses of the relation between ToM and children’s performance across different speech act categories. Results showed that both Age, ToM and their interaction could affect the children’s prosodic performance differently depending on the speech act category. The full estimates of all models are also provided in Table 3. Age was a significant predictor of performance for basic expressive acts (β = 0.122, *t* = 2.243, *p* = 0.027), suggesting that there is a gradual improvement in the expression of basic expressive acts of young preschoolers. ToM was not found to be a significant predictor of prosodic performance for any speech act, which suggests that ToM alone is not sufficient to explain the variation in prosodic scores. The interaction between Age and ToM was only significant for unbiased speech acts (β = 0.128, *t* = 2.917, *p* = 0.004). It turned out that higher ToM scores were positively related to better prosodic performance for unbiased items only in older children (β = 0.009, *t* = 3.832, *p* < 0.001). Yet within the younger group, children with higher ToM scores did not perform significantly better on prosody than children with lower ToM scores.

## Discussion and Conclusion

The main aim of the present study was to investigate the use of prosody by Catalan-speaking 3- to 4-year-old children for the expression of diverse pragmatic meanings. Results from the APT administered to 102 Catalan preschoolers have allowed us to sketch out a pragmatic prosody profile of children at this point in development by identifying the pragmatic uses of prosody according to speech act type that they have begun to acquire in an adult way and those that are still to be developed. Finally, by combining APT-derived data with ToM assessments, we have been able to assess the potential links between the development of pragmatic prosody skills and ToM at this age.

First, the results of the perceptive prosodic ratings (i.e., prosodic appropriateness scores given to children’s APT responses) showed that Catalan-speaking preschoolers deal well with the prosodic expression of basic pragmatic meanings such as basic expressive (e.g., greetings, calling) and unbiased speech acts, that is, unbiased requests (e.g., commands) and unbiased assertions (e.g., declarative statements with no biased meanings). However, they have more trouble in adequately producing prosodic cues related to the expression of pragmatic biases such as information structure (e.g., corrective/contrastive focus), belief states (e.g., incredulity, uncertainty, obviousness) and negation. All in all, these results corroborate previous findings on intonational development. For instance, previous work has shown that by 3 years of age children can use pragmatically appropriate prosody for basic speech acts. As mentioned above, Prieto et al. (2012, for Catalan and Spanish) and Frota et al. (2016, for Portuguese), showed that by the age of two children use an adult-like basic phonological inventory of nuclear pitch accents and boundary tones. This paper has shown that preschoolers employ a wide range of intonational contours and, more broadly, prosodic strategies, in order to express different types of pragmatic meanings, and that they do this with different degrees of competence depending on the specific pragmatic area involved.

As for the ability to express more complex pragmatic meanings, our findings confirm and expand the results from previous studies. For example, we found that 3- to 4-year-old children start to successfully express focus in biased assertions, producing appropriate pragmatic answers 37% of the time and 15% of appropriate prosodic answers. This rate contrasts with the results on unbiased assertions, where 74% of responses were pragmatically appropriate (33% of appropriate prosodic answers). This is consistent with Chen’s (2018) typological study suggesting that at 3 to 6 years of age children only begin to use intonational means for focus marking. As for the acquisition of epistemic prosody, our results showed that requests conveying epistemic meanings only obtained 24% of appropriate pragmatic responses (16% of appropriate prosodic answers). Even fewer appropriate responses (12%) were obtained for assertions expressing epistemic meanings (3% of appropriate prosodic answers). These results confirm previous studies on Catalan language indicating that young preschoolers already start to comprehend and express certain epistemic states through prosody (see, for example, Armstrong et al., 2018 and Hübscher et al., 2019b, on the comprehension of disbelief and the expression of uncertainty respectively) and that this ability improves with age. Focusing now on the production of a specific epistemic stance like uncertainty, Hübscher et al. (2019b) described different prosodic and gestural markers used by preschoolers to express uncertainty. For example, they reported that 3- to 4-year-old children extensively used rising uncertainty pitch contours. In our corpus, however, no felicitous examples of prosodic contours conveying uncertainty were obtained. A possible explanation for the differences between these findings could lie in the tasks used to elicit uncertainty expressions in preschoolers. While Hübscher et al.’s (2019b) user-friendly guessing game was probably cognitively easier for children, the APT required children to understand a set of diverse situational prompts, which is possibly a more challenging task for children.

In general, it is relevant to draw a parallel between the timeline in the acquisition of pragmatic prosody and the development of other pragmatic cues like morphosyntactic or lexical markers. For example, it has been shown that even though 4-year-olds start to change word order to mark focus (Sauermann et al., 2011), the ability to express information structure through syntactic cues is not yet acquired in the preschool years and it is not until middle childhood that children use syntactic strategies in an adult-like manner (Narasimhan and Dimroth, 2008; Dimroth and Narasimhan, 2012; Arnhold et al., 2016). Similarly, previous research has shown that children begin to express epistemic states through lexical markers (e.g., verbs “know,” “think”) at around the age of 3 (Diessel and Tomasello, 2001) and around the age of 4 they learn modal auxiliaries (e.g., “might,” “may”) (Papafragou, 1998; see also Matsui, 2014, for a review). Further research is needed to develop a full picture of the route of acquisition of different means that mark these pragmatic meanings and to establish the time window in which different focus and epistemic markers appear in children’s production, as well as to evaluate a potential precursor role of prosody in this respect. In order to gain a deeper understanding of the relationship between pragmatic and prosodic abilities, future studies investigating the two abilities through two separate tasks would be needed. In this view, it is of interest to compare our results with a recent study by Castillo et al. (in press) that reported a positive correlation between expressive pragmatic and prosodic abilities, where the latter were measured through an independent prosodic imitation task.

Second, our study also contributes to the relatively underexplored research area that investigates the link between ToM and prosodic skills. Though previous research has suggested that the development of receptive prosody is linked and constrained by ToM (for results in children, see Armstrong and Hübscher, 2018 and Armstrong et al., 2018; for results in adults, see Esteve-Gibert et al., 2020), as well as by other cognitive factors such as executive functions (Filipe et al., 2018; Ito, 2018), it should be noted that ToM measures are not usually included in studies on prosodic development. The present study examined the production side of pragmatic prosody and its link with ToM abilities. Our results suggest that ToM is not sufficient to explain and predict pragmatic prosody performance in preschool aged children, nor is the interaction between ToM and age. These results are in line with previous studies on prosodic skills in children with ASD (Chevallier et al., 2011; Colich et al., 2012) that showed that ToM impairment does not affect receptive prosodic abilities and extend them to expressive prosodic skills in typically developing children. Future research will need to gain a more in-depth understanding of how pragmatic prosody is acquired and determine the role of linguistic, socio-cognitive and age factors in that process. It might well be that a complementary set of individual variables may explain the development of pragmatic prosody skills. For instance, children’s core language skills such as vocabulary and syntax abilities, as well as their general socio-cognitive development as manifested, for example, in their ability to understand emotions and their comprehension of metacognitive vocabulary.

In relation to ToM effects, the pattern of results was also affected if different pragmatic skills were considered separately. Interestingly, our study found that the children’s performance on unbiased meanings was explained by the interaction between their age and ToM. These results indicate that the level of ToM does not equally affect children’s prosodic performance across ages. Specifically, higher ToM scores were positively related to better prosodic performance in older children (i.e., one standard deviation above the mean), while higher ToM scores were not associated with better prosodic performance in younger children (i.e., one standard deviation below the mean). This is most likely due to the floor effect observed for ToM in the younger group since younger children’s scores clustered toward the bottom end of the spectrum, and therefore their ToM scores did not vary much. By contrast, more variability was found in their prosodic performance, as the APT includes a variety of items targeting unbiased pragmatic meanings. It could be that older preschoolers’ (*M*_*age*_ = 45.12 months, *SD* = 3.27 months) developing understanding of others is manifested in the way they act in social environments and react to it using appropriate prosodic strategies (see also Hughes and Leekam, 2004). Unbiased pragmatic meanings presented in the APT embrace a wide range of common social situations that preschool children are likely to experience in their day-to-day life (e.g., answer an adult’s request, request some information, ask for a permission, produce a command, etc.). Thus, the explanation behind the finding of the role of ToM and age for children’s prosodic performance in unbiased pragmatic meaning relates to the claims of the literature that states the importance of social interactions for the children’s development of ToM (Carpendale and Lewis, 2004).

While the number of prosodically appropriate answers given to the items containing unbiased pragmatic meanings was predicted by the interaction between ToM and age, this was not the case with other pragmatic types. In particular, no link was found between epistemic prosody skills and ToM. This result contrasts with past research on prosodic aspects of the understanding of epistemic states showing that children’s comprehension of prosodically encoded disbelief is predicted by their ToM level (Armstrong et al., 2018). Yet our results go in line with previous studies on the more general relationship between the acquisition of epistemic meanings and ToM that found that children’s ability to understand and reason about epistemic concepts is not related to ToM (e.g., Tardif and Wellman, 2000; Perner et al., 2003; Tardif et al., 2004). As for focus, this study did not point to the role of ToM in the development of prosodic focus marking. Focus expression requires some cognitive flexibility, which is crucial for ToM (Jacques and Zelazo, 2005) as it relies on the ability to flexibly shift between conflicting perspectives. However, it could be that other (socio) cognitive factors can better explain the gradual acquisition of focus prosody. For example, focus prosody could be linked more to such cognitive resources as memory and attention span, since focus structures represent complex discourse structure and require children to switch attention between different referential elements. Therefore, in line with what has been previously suggested by Ito (2018), the ability to process and express complex information structure may be related to executive functions rather than to the ability to embed one’s perspective within other perspectives.

Overall, this is the first study that provides a comprehensive pragmatic prosody profile of 3- to 4-year-old Catalan speaking children. This prosodic profile could be established by using the APT test, which has been designed from children aged 3 until late childhood (see Pronina et al., 2019). In general, the children of the lower end of the age spectrum of the APT (3- to 4-year-olds) showed interest and engagement in the activity, successfully passed the familiarity items and understood the format of the task. Forty-two percent of the children were able to finish the test, which allowed us to gather a considerable amount of data on the prosodic patterns produced by preschool children while controlling for pragmatic contexts (i.e., a total of 905 prosodically appropriate responses). We believe that the APT has the potential to be a very useful tool in the field of developmental research because it allows researchers to elicit comparable semi-spontaneous speech data across individual children and child populations. Future studies might use the APT to build comprehensive profiles of developmental patterns in pragmatic prosody including older children and track the pragmatic prosody profile throughout childhood.

This study has several limitations. First, even though the use of the APT with 3-year-old children was successful, it revealed some shortcomings. As we have noted, 58% of the children were not able to fully complete the task (35 items). This might be explained by the fact that the APT is quite long and requires concentration on the part of the children. We should bear in mind that the APT was designed for a wide age span of children allowing researchers to track the acquisition of different pragmatic areas across development, that is why it could be possible that the youngest group (3–4 years) does not necessarily respond to all items of the test. While older children can successfully complete all the test (e.g., specifically, 94% of the 5- to 6-year-olds and 100% of the 7- to 8-year-olds finished the whole APT with 47 items, Pronina et al., in preparation), this was not the case with 3-year-olds. In line with the guidelines for developmental test batteries (e.g., see Semel et al., 2004; Carrow-Woolfolk, 2017), if during the administration of the APT it becomes clear that the test is too difficult or demanding for the child, the testing should be stopped and this does not mean a failure in the test (in our case, the child managed to pass familiarization items, engaged in the activity and answered as many items as (s)he could). Yet perhaps in order to increase the number of 3-year-old children fully completing the test it could have been better to shorten it or present the items in two separate sessions. Second, different speech acts were not represented equally in the APT (only first the 35 items were administered); it included 1 item of unbiased assertions, 7 items of biased assertions, 10 items of unbiased requests, 5 items of biased requests, 7 items of basic expressive acts and 5 items of complex expressive acts. Though the distribution strongly builds on previous research, specifically, as a whole, it is based on widely used standardized pragmatic developmental tests for children (e.g., Carrow-Woolfolk, 2017) and some pragmatic areas were enriched with additional items adapted for children from adult Discourse Completion Task questionnaires on Catalan prosody, this still entails that some pragmatic areas had more coverage more within the APT. Third, the use of the Discourse Completion Task (as opposed to more ecologically valid tasks) might have affected the results, which will need to be compared in the future with studies using other tasks. In this regard, the fact that some age discrepancies appear between our results and Hübscher et al.’s (2019b) study on the production of uncertainty prosody suggests that the choice of situational prompts for every pragmatic area may have affected the specific results on the developmental path. Future studies using more ecological tasks are therefore needed to help broaden our understanding of the acquisition of pragmatic uses of prosody across languages. Finally, in the present study prosodic abilities were estimated by perceptual judgments of Catalan raters. Future analyses of the data could perform more complete acoustic and prosodic analyses and focus on the typology of child-produced intonational pitch contours, as well as on their developmental patterns. Further analyses could also assess the development of the target intonational pitch contours produced by Catalan children by means of the ToBI coding system (see Prieto, 2014, for Cat_ToBI) and complementary acoustic analyses.

In conclusion, the comprehensive assessment of the acquisition of pragmatic prosody by young preschoolers (3- to 4-years of age) reported in this article demonstrates the importance of bridging the gap between prosody and pragmatics when accounting for prosodic developmental profiles, as well as of the relation of the acquisition of prosody to other developing abilities such as ToM. Our results shed light on the pragmatic prosody profile of young preschoolers, who are able to perform prosodic patterns of unbiased speech acts and have more trouble with prosodic expressions of pragmatic biases. Along these lines, we suggest that clinical prosodic instruments should include a more exhaustive assessment of speech prosody and look to integrate a wider range of its pragmatic functions. Moreover, the present findings also help elucidate the relationship between the acquisition of pragmatic prosody and cognitive capacities such as ToM. In general terms, by exemplifying the value of incorporating pragmatic abilities in prosodic assessment, this study underlies the importance of considering other linguistic and socio-cognitive dimensions in order to gain a more fine-grained picture of the acquisition of pragmatic prosody. At the same time, on a more practical plane, it suggests that a thorough evaluation of children’s pragmatic prosody profile could have diagnostic relevance in detecting pragmatic deficits.

## Data Availability Statement

Publicly available datasets were analyzed in this study. This data can be found here: https://osf.io/t6z3n/?view_only=bec881a58f68454f83200334cda2fad9.

## Ethics Statement

The studies involving human participants were reviewed and approved by The Ethics Committee at the Universitat Pompeu Fabra. Written informed consent to participate in this study was provided by the participants’ legal guardian/next of kin.

## Author Contributions

All authors participated in conceptualizing and designing this study as well as preparing materials. MP, IH, and PP organized the recruitment of the participants. MP collected and analyzed the data, and wrote the draft of the manuscript. PP contributed to interpreting and discussing the data and helped drafting the manuscript at different stages. All authors reviewed and contributed to revised drafts of the manuscript.

## Conflict of Interest

The authors declare that the research was conducted in the absence of any commercial or financial relationships that could be construed as a potential conflict of interest.

## Footnotes

1 https://osf.io/pyc34/?view_only=8c0f7d70c50a4dc1b01614c6b6521fb0

2 The two tests presented in this article form part of a bigger project on the development of pragmatic skills and their relation to other linguistic and socio-cognitive abilities (Pronina et al., submitted, 2021).

3 We subjected a 50% sample of our data to examine these zero scores. This analysis showed that in the majority of cases (88%) items were scored zero because the child made no attempt to answer the item, either by saying “I don’t know” or by simply remaining silent, or they commented on the pictures and situations without paying attention to the item in question. Only in 12% of cases did the children respond to the prompt but in a way that was pragmatically inappropriate [for example, this was the case if, for the item intended to elicit a farewell, the child responded by saying “Hola!” (“Hello!”)].
